# Study of Erythrocyte Indices, Erythrocyte Morphometric Indicators, and Oxygen-Binding Properties of Hemoglobin Hematoporphyrin Patients with Cardiovascular Diseases

**DOI:** 10.1155/2017/8964587

**Published:** 2017-07-17

**Authors:** Victor V. Revin, Antonina A. Ushakova, Natalia V. Gromova, Larisa A. Balykova, Elvira S. Revina, Vera V. Stolyarova, Tatiana A. Stolbova, Ilya N. Solomadin, Alexander Yu. Tychkov, Nadezhda V. Revina, Oksana G. Imarova

**Affiliations:** ^1^Federal State-Financed Academic Institution of Higher Education “National Research Ogarev Mordovia State University”, Saransk 430005, Russia; ^2^GBUZ RM “National Hospital for War Veterans”, Saransk 430005, Russia

## Abstract

The current study investigates the functional state of erythrocytes and indices of the oxygen-binding capacity of hemoglobin in blood samples from healthy donors and from patients with coronary artery disease and myocardial infarction before and after treatment. It has been established that, in cardiovascular diseases, erythrocyte morphology and hemoglobin oxygen-transporting disorders are observed. Standard therapy does not result in the restoration of the structure and properties of erythrocytes. The authors believe that it is necessary for future therapeutic treatment to include preparations other than cardiovascular agents to enhance the capacity of hemoglobin to transport oxygen to the tissues.

## 1. Introduction

Currently, cardiovascular diseases are the most common diseases and are one of the leading causes of death and disability among able-bodied populations in economically developed countries [1–3]. By 2020, the World Health Organization estimates that there will be nearly 25 million deaths due to cardiovascular diseases worldwide.

More than half of deaths due to cardiovascular system diseases are caused by coronary artery disease (CAD). One in five men aged from 50 to 59 years suffers from this disease, and the incidence and mortality rates are increasing every year.

Coronary artery disease may cause myocardial infarction, apoplectic attack, or heart failure. Acute heart failure (AHF) remains one of the most actual and important problems of modern cardiology. Acute heart failure is a clinical syndrome characterised by early onset of disturbed cardiac function symptoms (reduced cardiac output and inadequate blood supply) [4, 5]. At present, the most common causes of acute heart failure are myocardial infarction (MI), decompensated chronic heart failure (CHF), and heart rhythm disorders, including atrial fibrillation [5].

Abnormalities of blood rheology are of great importance among significant factors determining hemodynamic disorders found in patients with CAD [6–8].

Among other factors underlying the pathogenesis of CAD (coronary atherosclerosis, hemodynamic findings, and coagulation system imbalances), hemorheological disorders influence CAD severity, expected treatment response, and patient treatment success [8–10].

Retrogressive changes in hemorheological indices are closely related to changes in erythrocyte morphometric indicators, which, in turn, determine their functional properties and reflect the state of both erythrocyte and cell membranes. Moreover, changes in erythrocyte morphometry may serve as an indicator of conduct therapy efficiency.

Hypoxia plays a significant role in the pathophysiology of a majority of cardiovascular diseases. Hypoxia causes disorders of blood gas transport function and very often leads to decreases in the efficiency of oxygen transport with the assistance of erythrocytes. In such cases, disorders of endothelial structure and function play a crucial role, whereas the role of erythrocytes and their oxygen-transporting capacity in the progress of peripheral vascular diseases still remains under investigated [3, 11].

One of the main reasons for disorders of the erythrocyte oxygen-transporting function is the conformational change of hemoglobin hematoporphyrin (Hb) and hemoglobin oxygen affinity (О_2_). The use of Raman scattering spectroscopy makes it possible to determine changes in the conformation of hematoporphyrin in patients with severe arterial hypertension [12], patients with circulatory failure [13–15], and patients with stable effort angina under therapeutic interval hypoxia [12]. It is also important that the oxygen-binding and oxygen-transporting properties of hemoglobin depend on the morphofunctional state of erythrocyte membranes [16, 17].

A change of the conformation of hemoglobin hematoporphyrin (both cytoplasmic and membrane-bound) is may be interconnected with changes in the morphometric indicators of erythrocytes (erythrocyte surface area, phase height, phase volume, and hemoglobin distribution). Erythrocyte indices are an additional characteristic of the morphological and functional properties of erythrocytes.

The purpose of this study was to investigate erythrocyte morphology, the functional state of erythrocytes, and the conformational changes in hemoglobin in both healthy donors and patients with cardiovascular diseases.

To achieve our designated research purpose, the following aims were set:

1. To study the functional characteristics of erythrocytes and the state of hemoglobin healthy donors and in patients suffering from CAD with various levels of severity for angina of effort and MI on their admission to hospital
2. To evaluate the changes in the functional characteristics of erythrocytes and the state and oxygen-binding capacity of hemoglobin in patients with CAD and other cardiovascular diseases after standard medical treatment

## 2. Materials and Methods

### 2.1. Patients

This study included 40 patients from a cardiovascular care unit (Veterans Hospital of the Republic of Mordovia). Patients were males aged from 41 to 60 years (average age is 53.6 ± 4.2 years).

A clinicofunctional analysis of the condition of 20 patients suffering from CAD with stable angina (SA) of effort of the functional classes II-III was carried out.

Patients selected for subsequent examination did not smoke, had no genetically determined diseases, and had body mass indices ranging 24–29.

Twenty male patients diagnosed with “acute myocardial infarction” were also examined.

Therefore, all patients were divided into 2 groups based on a disease:

  Group 1: stable angina, *n* = 20
  Group 2: acute myocardial infarction, *n* = 20

Treatment included standard methods of clinical research study. All patients provided their consent to participate in the study.

The patients were randomly selected, and patients older than 60 years were excluded. The research was assessed by experts and approved by Local Ethics Committee at the Mordovia State University. The research was conducted in accordance with the Good Clinical Practice principles.

### 2.2. Control Group

There was also a control group consisting of 20 practically healthy donors (aged 41–60 years) who had periodic health examinations and did not have a history of cardiovascular diseases. These donors were selected in such a way that their gender and age corresponded to the parameters of patients with heart diseases from the other studied groups. Their average hematological parameters corresponded to physiologically normal parameters typical for such gender and age.

### 2.3. Sample Collection

Patient blood samples were taken from patients with diseases in the morning before taking medications or consuming food. Samples were taken from the ulnar vein and were collected into 5 ml vacuum tubes. The blood samples were taken from patients at their admission to hospital and after the protracted treatment based on the background therapy. The standard therapy in case of cardiac angina (CA) and MI included the following: regimen, diet, intake of statins (atorvastatin С_33_H_35_FN_2_О_5_), ACE inhibitors (enalapril С_20_H_28_N_2_О_5_) or blockers of the angiotensin receptor (valsartan С_24_H_29_N_5_О_3_), beta-blockers (bisoprolol NС_18_НNО_4_), antiaggregants (if CA, aspirin С_9_Н_8_О_4_; if MI, additionally, clopidogrel C_16_H_16_ClNO_2_S), if MI, anticoagulants (heparin C_12_H_19_NO_20_S_3_), and nitrates (nitroglycerin) on demand. On medical indications, in case of MI, we administered analgesics (morphine), diuretics (verospiron), antiarrhythmic medication, and cardiac tonics. As for the group with MI, our study included patients who were delivered stenting (bare metal stents).

Thus, all 100% of CA patients took aspirin, and MI patients received 3-component therapy: aspirin + clopidogrel + heparin during 7–10 days under the control of APTT.

Blood was taken according to a standard procedure of blood taking [18].

For complete blood counts (CBC), vacuum systems with K3-EDTA anticoagulant were used. Rосhе Diagnostics (Switzerland) reagents were used in addition to dilution reagent (Сеllрасk), lysing solution (Stromatolyser 4DL, Sulfolyser), leucocyte differentiation reagent (Stromatolyser 4DS), and caustic cleaner (Сеllсlеаn).

### 2.4. Hematologic Research

Hematologic research was conducted by means of an ХT-2000i automatic hematology analyser (Sysmех, Japan). For hematological indices, we determined the following indicators: erythrocyte levels (RBC count), mean corpuscular volume (MСV), mean corpuscular hemoglobin (MСH), and mean corpuscular hemoglobin concentration (MСHС).

### 2.5. Raman Analysis

The oxygen-binding properties of hemoglobin were evaluated by examining changes in the conformation of hemoglobin hematoporphyrin in native erythrocytes by means of Raman spectroscopy using an inViа Raman-scattering spectrometer (Renishaw, United Kingdom) with a short focal distance and high transmission monochromator with a focal distance no longer than 250 mm. A laser was used to obtain spectra (radiation wavelengths: 532 nm; peak radiated power: 100 mW; field lens: 100x). For data recording, a CCD detector (1024 × 256 pixels with a Peltier cooling module maintaining it at −70°С) with a grating of 1800 lines per mm was used. Digitised spectra are adapted using the WiRE 3.3 program. Spectral smoothing and baseline correction were also performed.

Whole blood was centrifuged during 10 min at 4000*g*. After removing the supernatant liquid, the sediment of erythrocytes was washed 10-fold volume of the solution for washing the erythrocytes and then again centrifuged 10 min at 4000*g* (at a temperature of 4°С). The above-described procedure was repeated 3 times. The obtained erythrocytic mass was stored in the washing medium at 4°C. Time of storage did not exceed 1 hour.

Smear was prepared on the slide (glass) by a standard method (slide was degreased with alcohol and dried prior to smearing), immediately after preparation measurements were taken of spectra of hemoglobin hematoporphyrin of erythrocytes (in the range 300–2000 sm^−1^).

To evaluate the conformation of hemoglobin hematoporphyrin (Hb) and hemoglobin oxygen-binding properties, specific Raman-scattering spectral lines were used (maximum values are given): 1355, 1375, 1550, and 1580 cm^−1^.

The nature of the Raman spectra for hemoglobin hematoporphyrin [19] allows one to identify the degree of oxidation of its iron atom, its spin state, and the existence of ligands. It also reflects changes in globin structure that lead to hematoporphyrin deformation and influencing the oxygen-binding properties of hemoglobin [14].

The intensities of the 1355 and 1375 cm^−1^ spectral lines are connected with symmetrical oscillations of pyrrole rings in molecules of deoxygenated hemoglobin and hemoglobin and with ligands, respectively [13]. The intensity of the 1375 cm^−1^ line is determined by oxygenated hemoglobin contents because the amount of oxygen in blood is 3-4 times higher than the contents of other ligands (e.g., NO or CO). Thus, the intensity ratio *I*_1375_/(*I*_1355_ + *I*_1375_) is proportional to the relative amount of oxygenated hemoglobin in blood. Intensities of the 1550 cm^−1^ and 1580 cm^−1^ spectral lines characterise the spin state of iron in deoxy- and oxy-forms, respectively. Therefore, they act as markers to evaluate the structural characteristics of iron in a prosthetic group, which provides the opportunity to use the spectral line intensity ratio* I*_1355_/*I*_1550_ and* I*_1375_/*I*_1580_ to evaluate the capacity of hemoglobin molecules in erythrocytes to bind and donate oxygen molecules, given the internal state of hemoglobin molecules. After division of one ratio by another (*I*_1355_/*I*_1550_)/(*I*_1375_/*I*_1580_), it is possible to obtain the characteristic reflecting hemoglobin molecule oxygen affinity in native erythrocytes [20, 21].

### 2.6. Laser Interference Microscopy

Morphometric indices (morphology) were studied by laser interference microscopy. In contrast to the conventional methods of optical microscopy based on the registration of light intensity distribution, laser interference microscopy allows one to obtain the phase distribution in the interference image [22, 23].

For this study, an MII-4M modulation interference microscope, built by “Amphora Laboratory” (Moscow, Russia), was used. We also used a 635 nm laser with a lens with a magnification of 33.4 diameters and a numerical aperture of 0.65 [24]. A preliminary calibration of the microscope using 2 *µ*m and 7.5 *µ*m silicone particles was performed. Using a laser and interference microscope, the following morphological properties of erythrocytes were determined: the optical path difference (OPD), the surface area of the erythrocyte phase image (*S*), the physical height (*Z*), and phase volume of erythrocytes (*V*) [25].

The principle underlying the operation of this device is as follows: a laser beam *L* is divided into reference (control) and object beams. The reference beam reflects off the inspection mirror and goes to the detector. The object beam passes through the object, which is placed into a special chamber with the mirror bottom, reflects off the mirrored base sheet, passes through the object again, and goes to the detector, where it interferes with the first beam. Due to the difference in refractive indices of environment and the object between beams, there is optical path difference or phase height.

OPD value search point of the object forms phase image of the object [26–28]. The phase image for all the cells depends on the refractive index distribution and geometrical size of the cell. If geometrical size of the cell does not change during the experiment, then changes of phase height are determined only by changes of the refractive index. Thus, by the phase image, we can judge refractive index distribution in the cell, by the OPD change, the refractive index dynamics, and hence intracellular processes [29].

We placed 5–10 *µ*l of erythrocytic suspension diluted with normal saline at a ratio of 1 : 200 onto special preparation glass with a smooth surface, and the sample was then covered with glass and placed under the microscope lens.

The mirrored base sheet on which the preparation with erythrocytes was placed reflected transmitted light, resulting in a double-phase shift of the coherent light source beam at every point on the object, and by means of an additional wave from the same source, the interference cell image was formed. At least 100 cells were imaged for each test [22, 25].

Interference images processing was carried out using FIJI software [30]. Using this program, the registration of the surface area volume of erythrocytes and maximum path length difference were performed. Further analysis of the results was carried out using Microsoft Office Excel 2013 and OriginPro 8.1 programs.

We calculated the erythrocyte phase volume using the following formula:

(1) $$\begin{array}{c} V=\frac{{Ф}_{mean}*S}{n_{cell}-n_{m}}. \end{array}$$

We calculated physical height using the following formula:

(2) $$\begin{array}{c} Z_{cell}=\frac{{Ф}_{mean}}{n_{cell}-n_{m}}. \end{array}$$

 
*Ф*_m*еа*n_ is mean value of measured parameter of the optical path length difference, proportional thickness.
 
*S* is surface area of the phase image of the cell.
 
*n*_cell_ is erythrocyte refractive index, which is 1.405.
 
*n*_*m*_ is refractive index of the surrounding solution (normal saline), which was 1.333 [25].

Refractive index was measured by using the Refractometer PTR46 [31–33].

Therefore, the erythrocyte phase portrait forming the phase shift distribution in various parts of the object was obtained. Phase shift values were used to create a three-dimensional (3D) image of the cell.

### 2.7. Statistical Analysis

At the first stage of statistical analysis, we evaluated the normality of value distribution for every sample using Geary's criterion [34]. We then evaluated the homogeneity of variance. We conducted the analysis of variance model and ANOVA for repeated measurements. In case of statistically significant differences between average values, we used ex post facto Tukey's method to compare individual means analysis [35].

The findings are presented in the form of arithmetical mean and standard deviation (mean ± SD).

## 3. Results 

### 3.1. RBC Count and Erythrocyte Indices in the Blood of Apparently Healthy People and in Patients with SA and MI before and after Treatment

Erythrocyte indices are calculated values that allow for the quantification of important indicators of the erythrocyte state. These indices include the total number of erythrocytes, MСV, MСH, and MСHС. These indices are used to assess the functional activity of erythrocytes. For example, the MСHС is a sensitive indicator of hemoglobin formation violations, as it shows the degree of hemoglobin saturation in erythrocytes regardless of the amount of formed elements. The MCH indicator (mean corpuscular hemoglobin) estimates the weight of hemoglobin in an erythrocyte or the hemoglobin ratio to the cell volume.

Haemogram assessment has been based on physiological standards corresponding to the international system of units (SI) in clinical studies [36].

We found that the red blood cell count of apparently healthy people was 4.47 ± 0.21 × 10^12^ cells/L, the mean corpuscular volume was 94.51 ± 4.41 fL, and the mean corpuscular hemoglobin and the mean corpuscular hemoglobin concentrations were 31.17 ± 1.17 рg and 343.3 ± 8.23 рg, respectively. These indices that do not differ from physiological standards and literature data have been used as controls in our research (Table 1).

The data of patients with SA and MI admitted to the hospital were analysed before treatment and after the patient's hospital discharge.

At the time of hospital admission, the red blood cell counts of patients with CAD corresponded to physiological standards, although they exceeded the control group indices by 8% (Table 1).

Erythrocyte indices in patients with IHD and with MI corresponded to physiological norms. At the same time, all indices in the patient groups were significantly lower than those of the control group of donors.

Statistically significant changes in the studied indices were not observed after treatment.

Qualitative and quantitative changes, observed in the red blood cell system, are important diagnostic and prognostic indicators of various pathological processes and diseases. However, RBC indicators and erythrocyte indices provide only an indirect understanding of the processes occurring in the cytoplasmic membrane of red blood cells and can show not only the degree of erythrocyte damage (change in the morphology of each erythrocyte) but also the state (conformation and form) of hemoglobin in the erythrocyte.

Since the RBC count, MСV, MСHС, and MСH of patients with SA and MI were within normal physiological standards, we could not estimate real changes in the morphology of each erythrocyte. In addition, it was difficult to understand which hemoglobin form (oxyhemoglobin, deoxyhemoglobin, and methemoglobin) is within the erythrocyte and how the hematoporphyrin conformation and the hemoglobin oxygen-binding capacity change.

In this manner, we studied the morphometric characteristics of red blood cells using laser interference microscopy (LIM).

### 3.2. Morphometric Characteristics of Erythrocytes from Apparently Healthy People and from Patients with SA and MI before and after Treatment

The mean value of the optical path difference (OPD), the phase image area of erythrocytes (*S*), the geometric height (*Z*), and the erythrocyte phase volume (*V*) was estimated in patients with SA and MI.

Patients with CAD had erythrocyte morphometric characteristics that differed from that those of healthy people (Table 2).

The ОРD and geometric height of erythrocytes exceeded the control group indices by 17%, whereas the erythrocyte phase volume was 9.8% greater than that of controls. In addition, we noted a decrease in the erythrocyte phase image area in relation to controls by 6.5%. MI patients, upon admission to hospital, had a mean ОРD, erythrocyte geometric height, and erythrocyte phase volume that exceeded the indices of the healthy donors by 23.5%, 23.7%, and 15.9% (*p* < 0.05), respectively (Table 2). We noted a decrease in the erythrocyte phase image area in relation to the control by 6.4%.

After the treatment of patients with SA, we observed a decrease in the OPD and physical height by 35% and a decrease of the erythrocyte phase volume by 21.5% compared to the primary indices (*p* < 0.05). However, none of these indices ever returned to the control group levels.

After patients with MI were treated, their mean optical path difference and geometric height values barely changed, whereas their phase volume decreased by 27% compared to their indices at admission. Additionally, the phase image area of erythrocyte increased by 8%, significantly exceeding healthy donor group indices. Erythrocyte phase volume indices did not return to those of the control group.

We have made a correlation analysis between the morphometric parameters determined by the LIM method and values of erythrocytic indexes. When studying correlations between RBC values (red blood cell count) and LIM parameters of human erythrocytes, we detected a moderate negative correlation with the value of phase image *S* (*r* = −0.40) and moderate positive correlation with the values of the geometric height, phase volume, and optical path difference (*r* = 0.47, *r* = 0.48, and *r* = 0.48, resp.) (*p* < 0.05).

Moderate reverse correlation between MCH (mean hemoglobin content in the erythrocyte) and such LIM parameters like phase volume (*r* = −0.34), geometric height (*r* = −0.31), and OPD (*r* = −0.31) was discovered (*p* < 0.05).

The average correlation coefficient was also observed between MCV values and such morphometric parameters as OPD (*r* = −0.34), the phase volume (*r* = − 0.35), and geometric height (*r* = −0.34) (*p* < 0.05).

Between morphometric LIM values of human erythrocytes and the average concentration of hemoglobin in erythrocytes, MCHC correlation was not found.

**Figure 1:**
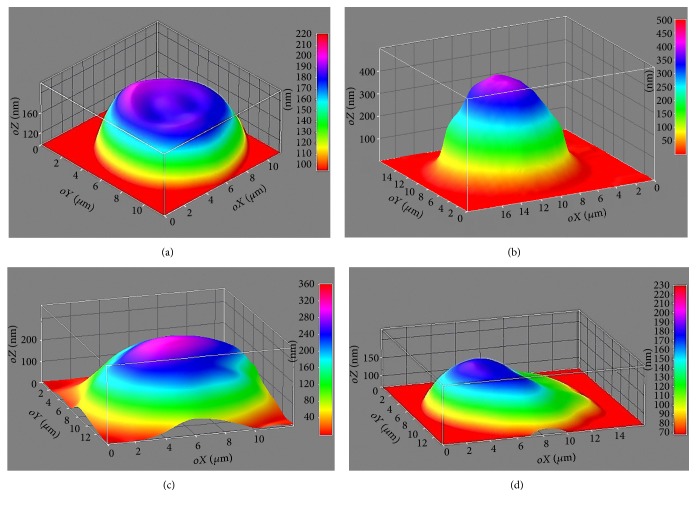
Phase images of erythrocytes, taken using LIM (*oX*, *oY*: erythrocyte sizes, *μ*m; *oZ*: optical path difference (OPD), nm): (a) erythrocyte from a healthy donor (discocyte); (b) erythrocyte from a MI patient before treatment (spherocyte); (c) erythrocyte from a SA patient before treatment (stomatocyte); (d) erythrocyte from a MI patient after treatment (stomatocyte).

Most erythrocytes from healthy people are represented by discocytes (Figure 1(a)), which is also shown by our data in the control group. SA and MI patients have had many more spherocytes among erythrocytes (Figure 1(b)). This erythrocyte class refers to the irreversibly deformed or prehemolytic forms [37]. Also stomatocytes met in large quantities (Figure 1(c)). Such changes in red blood cell shape in patients with cardiovascular diseases are primarily related to the violation of erythrocyte membrane stability [24, 38]. In the future, the output of ectoglobular hemoglobin (EGH) and its degradation products into blood plasma may be possible, which contributes to serious metabolic changes, secondary activation of lipid peroxidation, and exacerbation of ischaemic myocardial injury [39].

The number of spherocytes was reduced, whereas other pathological forms of erythrocytes related to reversible forms such as stomatocytes and echinocytes were more frequent (Figure 1(d)) in the patient group after the treatment. Compared with discocytes, degenerative forms of red blood cells are less robust in terms of oxygen delivery function, microcirculation, and deformation capacity; therefore, their increase is an unfavourable sign [37]. Their ability to aggregate depends on their factors too. Increased erythrocyte aggregation disrupts the normal structure of the blood stream in the microcirculatory vessels and leads to increased blood viscosity, microcirculatory blocks, and tissue hypoxia [40].

### 3.3. Correlation of Raman Bands of Erythrocyte Hemoglobin Hematoporphyrin of Apparently Healthy People and People with SA and MI before and after Treatment

**Figure 2:**
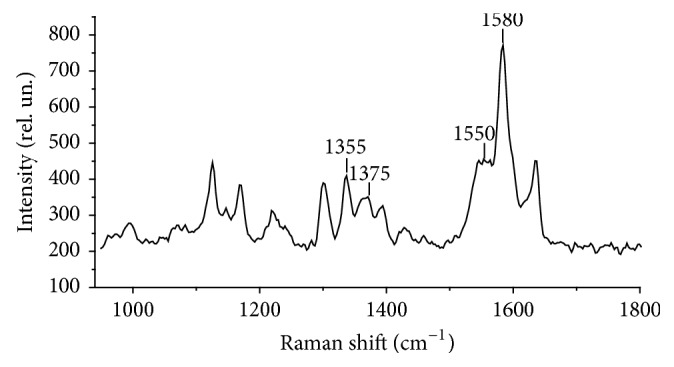
Spectrum of Raman scattering of hematoporphyrin hemoglobin. The figure shows the Raman bands with the position of the maximums of 1355, 1375, 1550, and 1580 cm^−1^. The ordinate is the intensity of the Raman radiation, in conventional units; along the abscissa-frequency shift, cm^−1^.

Using the Raman method, we examined scattered intensity shifts for the relevant spectral bands of erythrocyte hemoglobin hematoporphyrin from patients with SA and MI before and after treatment in relation to the control group. The conformation of hematoporphyrin hemoglobin of red blood cells, determined by the ratio of Raman spectra, of healthy donors is shown in Figure 2.

In cases of myocardial infarction and coronary artery disease, erythrocytes show changes in the correlation of band intensity.

The erythrocyte hemoglobin oxygen-binding ability indicators of patients with CAD at the time of hospital admission differed from those of healthy donors. Thus, the percentage of oxyhemoglobin in the blood was reduced by 21.8% and relative ability of hemoglobin to drop off ligands by 16.9% in comparison with the control group (*p* < 0.05). On the contrary, the relative ability of hemoglobin to bind ligands and hemoglobin ligand affinity exceeded the control level by 14.8% and 34.5%, respectively (Table 3).

After the treatment of patients with SA, the ability of hemoglobin to bind ligands did not reasonably change; it remained higher than that of the control group by 22.9% (*p* < 0.05) (Table 3). The ability of hemoglobin to allocate ligands has not changed. Compared to the control group, the index remained lower by 9.5% (*p* < 0.05). The hemoglobin ligand affinity was reduced by 13.5% (*p* < 0.05), although it did not reach the level of the control group. The percentage of oxyhemoglobin increased by 11.9% (*p* < 0.05), remaining lower than the control value (*p* < 0.05).

Before the treatment of patients with MI, the ability of hemoglobin to bind ligands and the hemoglobin ligand affinity increased by 24.3% and 64.8% relative to the control, and the ability of hemoglobin to shed ligands and the percentage of oxyhemoglobin decreased by 24.5% and 24.7%, respectively, compared to the control. After treatment, the ability of hemoglobin to bind ligands irrelevantly decreased, and the hemoglobin ligand affinity decreased by 9.0%; we also observed an increase of 11.4% in the percentage of oxyhemoglobin. Simultaneously, the ability of hemoglobin to allocate ligands did not change, remaining higher than the control value by 20.8%.

The studied indices still did not reach the control levels (Table 3). The changes in the study results are therefore better expressed among MI patients.

When studying correlations between the relative number оHb in the blood (*I*_1375_/(*I*_1355_ +* I*_1505_)) and morphometric parameters of human erythrocytes, we discovered a moderate negative correlation with values of the optical path difference (*r* = −0.46), geometric altitude (*r* = −0.46), and phase volume (*r* = −0.47). High correlation coefficient was observed with values of erythrocyte indices: RBС count (*r* = −0.72), MCV (*r* = 0.98), MCH (*r* = 0.95), and MCHC (*r* = 0.94) (*p* < 0.05).

When studying correlations between the relative ability of hemoglobin to release ligands (*I*_1375_/*I*_1550_) and morphometric parameters of human erythrocytes, correlation was not found.

When studying correlations with values of erythrocytic indices, we detected a moderate reverse correlation with RBC count (*r* = −0.44). With MCV, MCH, and MCHC indices, we found a close direct positive correlation (*r* = 0.88, *r* = 0.88, and *r* = 0.84, resp.) (*p* < 0.05).

During the study of correlations between the relative ability of hemoglobin to bind ligands, including oxygen (*I*_1355_/*I*_1550_) and the morphometric parameters of human erythrocytes, correlation was also not found.

We found a moderate direct correlation with the RBC index (*r* = 0.32) and a strong reverse correlation with MCV, MCH, and MCHC indices (*r* = −0.82, *r* = −0.83, and *r* = −0.78, resp.) (*p* < 0.05).

During the study of correlations between the affinity of hemoglobin to ligands, particularly to oxygen ((*I*_1355_/*I*_1550_)/(*I*_1375_/*I*_1580_)) and morphometric parameters of erythrocytes, we detected a close reverse correlation with such erythrocytic indices as MCV, MCH, and MCHC (*r* = −0.87, *r* = −0.92, and *r* = −0.85, resp.). A moderate direct correlation with OPD values, physical thickness (*r* = 0.51), and phase corpuscular volume (*r* = 0.53) was detected (*p* < 0.05).

## 4. Discussion

In the pathogenesis of SA and MI, the universality and severity of microcirculation disorders are highly significant [41]. The persistence of the microvasculature function is mostly determined by the rheological properties of blood [13]. Therefore, in the pathogenesis of SA and MI, rheological disorders are of great importance, and the participation of red blood cells in this process determined our interest in studying the structural and functional characteristics of red blood cells [42].

This study demonstrated that, in the case of cardiovascular diseases such as SA and MI, patient erythrocyte index values corresponded to physiological standards.

However, by using more subtle methods such as LIM and Raman spectroscopy, we discovered changes in erythrocyte morphology and the violation of the oxygen-binding ability of hemoglobin associated with conformational rearrangements of hematoporphyrin.

Therefore, cardiovascular diseases are followed by an increase in erythrocyte ОРD, physical height, and phase volume. Changes in erythrocyte morphological characteristics are directly related to irregularities in the composition of the phospholipid component of erythrocyte membranes and an increase in their microviscosity [43].

It is known that CAD patients have increased erythrocyte plasma membrane viscosity [44]. At the same time, oxyhemoglobin levels reduce due to the deterioration of oxygen diffusion through the membrane and the decrease of the oxygen saturation of red blood cells, which contributes to tissue hypoxia.

An increase in hemoglobin oxygen affinity and the ability of hemoglobin to bind oxygen leads to additional increases in hypoxia processes that are already present at the development of SA and especially MI. This increase can also be caused by the deterioration of the ability of hemoglobin to shed ligands, including oxygen.

Patients with cardiovascular diseases undergo long-term drug therapy.

Among the most frequently used drugs in cardiology practice are medications containing nitrates. Currently, several medications are used: nitroglycerine (or glyceryl trinitrate), statins (Liprimar), ACE inhibitors (enalapril), beta-blockers (bisoprolol, Concor), blockers of angiotensin receptors (valsartan), and anticoagulants (heparin). Organic nitrates are donors of exogenous NO with physiological effects identical to exogenous NO. Their activity is shown after a series of metabolic transformations in which nitrogen oxide is formed, that is, a substance similar in structure and function to the endothelium-dependent relaxation factor (EDRF) [45]. The main goal of drug therapy is to decrease the venous vessel pressure. Anticoagulants prevent blood clot formation and intravascular coagulation. Statins have lipid-lowering effects and help to reduce LDL levels, which helps prevent the proliferation of atherosclerotic plaques and reduces the risk of strokes and heart attacks [46].

Unfortunately, such standard SA and MI therapies do not lead to the recovery of erythrocyte structure and the oxygen-binding ability of hemoglobin.

## 5. Conclusion

Cardiovascular pathology is associated not only with violations of the structure and function of the blood vessels endothelium but also with changes in the structure and oxygen-binding capacity of erythrocytes.

On the basis of our data and an analysis of the literature, we argue that, in addition to traditional drugs, which affect the status of the vessels, it is necessary to seek natural compounds that normalise the structure of red blood cells and enhance their oxygen transport function to more effectively treat patients with various cardiovascular diseases.

## Figures and Tables

**Table 1:** RBC count and erythrocyte indices in the blood of apparently healthy people and in patients with SA and MI before and after treatment, *n* = 20 (М  ± SD).

Test groups	Indices			
	RBС count (3.8–5.3) × 10^12^cells/L	MСV (80–100) fL (femtolitre)	MСH in one erythrocyte (27–32) рg (picogram)	MСHС (320–360) рg (picogram)
Control	4.47 ± 0.20	94.51 ± 4.41	31.17 ± 1.18	343.3 ± 8.23
SA patients before treatment	4.87 ± 0.41^*∗∗*^	88.37 ± 4.09^*∗∗*^	29.98 ± 1.06^*∗*^	330.6 ± 20.45^*∗*^
SA patients after treatment	4.75 ± 0.34	90.35 ± 4.81	30.09 ± 1.20	332.0 ± 3.66
MI patients before treatment	4.76 ± 0.31^*∗*^	88.3 ± 4.14^*∗∗*^	29.54 ± 1.63^*∗*^	329.3 ± 12.57^*∗*^
MI patients after treatment	4.52 ± 0.45	90.12 ± 2.74^*∗*^	30.22 ± 1.03	333.85 ± 12.29

^*∗*^ *p* < 0.05; ^*∗∗*^*p* < 0.01.

**Table 2:** Morphometric characteristics of erythrocytes from apparently healthy people and from patients with SA and MI before and after treatment, *n* = 20 (М  ± SD).

Test groups	Indices			
	The mean value of optical path difference (OPD), Φ_mean_, nm	Phase image area of erythrocyte, *S*, *µ*m^2^	Geometric mean height of the erythrocyte, *Z*, *µ*m	Erythrocyte phase volume, *V*, *µ*m^3^
Control	151.82 ± 6.46	43.43 ± 3.79	2.02 ± 0.11	87.73 ± 4.82
SA patients before treatment	177.39 ± 5.39^*∗*^	40.80 ± 3.30	2.36 ± 0.14^*∗*^	96.29 ± 3.55^*∗*^
SA patients after treatment	115.38 ± 4.61^*∗*Δ^	55.99 ± 2.93^*∗*Δ^	1.53 ± 0.11^*∗*Δ^	75.59 ± 3.99^*∗*Δ^
MI patients before treatment	187.53 ± 6.41^*∗*^	40.66 ± 2.90	2.50 ± 0.15^*∗*^	101.65 ± 2.98^*∗*^
MI patients after treatment	114.39 ± 3.82^*∗*Δ^	59.40 ± 2.25^Δ^	1.52 ± 0.14^*∗*Δ^	74.25 ± 4.46^Δ^

^*∗*^ *p* < 0.05, reliability in relation to donors' indicators; ^Δ^*p* < 0.05, reliability in relation to pretreatment indicators.

**Table 3:** Correlation of Raman bands of erythrocyte hemoglobin hematoporphyrin of apparently healthy people and people with SA and MI before and after treatment, *n* = 20 (М  ± SD).

Test groups	Indices			
	Percentage of oxyhemoglobin blood *I*_1375_/(*I*_1355_ + *I*_1375_)	Relative ability of hemoglobin to bind ligands *I*_1355_/*I*_1550_	Relative ability of hemoglobin to drop off ligands *I*_1375_/*I*_1580_	Hemoglobin ligand affinity (О_2_) (*I*_1355_/*I*_1550_)/(*I*_1375_/*I*_1580_)
Control	0.69 ± 0.03	0.61 ± 0.03	0.53 ± 0.02	1.16 ± 0.07
SA patients before treatment	0.54 ± 0.02^*∗*^	0.70 ± 0.03^*∗*^	0.44 ± 0.03^*∗*^	1.56 ± 0.12^*∗*^
SA patients after treatment	0.61 ± 0.03^*∗*^	0.67 ± 0.04^*∗*^	0.48 ± 0.03	1.41 ± 0.15^*∗*^
MI patients before treatment	0.52 ± 0.03^*∗*^	0.76 ± 0.03^*∗*^	0.40 ± 0.02^*∗*^	1.90 ± 0.11^*∗*^
MI patients after treatment	0.58 ± 0.04^*∗*^	0.75 ± 0.05^*∗*^	0.42 ± 0.03^*∗*^	1.52 ± 0.10^*∗*Δ^

^*∗*^ *p* < 0.05, reliability in relation to donors' indicators; ^Δ^*p* < 0.05, reliability in relation to pretreatment indicators.

## References

[1] Sasayama S. (2008). Heart disease in asia. *Circulation*.

[2] Jeemon P., Reddy K. S. (2010). Social determinants of cardiovascular disease outcomes in Indians. *Indian Journal of Medical Research*.

[3] Upadhyay R. K. (2015). Emerging risk biomarkers in cardiovascular diseases and disorders. *Journal of Lipids*.

[4] Gheorghiade M., Bonow R. O. (1998). Chronic heart failure in the United States: A manifestation of coronary artery disease. *Circulation*.

[5] Gheorghiade M., Pang P. S. (2009). Acute heart failure syndromes. *Journal of the American College of Cardiology*.

[6] Dintenfass L. (1985). Red cell aggregation in cardiovascular diseases and crucial role of inversion phenomenon. *Angiology*.

[7] Mares M., Bertolo C., Terribile V., Girolami A. (1991). Hemorheological study in patients with coronary artery disease. *Cardiology*.

[8] Bilgi M., Güllü H., Kozanoğluetal İ. (2013). Evaluation of blood rheology in patients with coronary slow flow or non-obstructive coronary artery disease. *Clinical Hemorheology and Microcirculation*.

[9] Kesmarky G., Toth K., Habon L. (1998). Hemorheological parameters in coronary artery disease. *Clinical Hemorheology and Microcirculation*.

[10] Rozanski A., Blumenthal J. A., Kaplan J. (1999). Impact of psychological factors on the pathogenesis of cardiovascular disease and implications for therapy. *Circulation*.

[11] Pierson D. J. (2000). Pathophysiology and clinical effects of chronic hypoxia. *Respiratory Care*.

[12] Maksimov G. V., Rodnenkov O. V., Churin A. A., Rubin A. B., Tkachuk V. A., Chazov E. I. (2001). Influence of interval hypoxemic training on hemoglobin ability to bind oxygen in the blood of ischemia heart disease patient. *Cardiology*.

[13] Rodnenkov O. V., Luneva O. G., Ulyanova N. A. (2005). Erythrocyte membrane fluidity and haemoglobin haemoporphyrin conformation: Features revealed in patients with heart failure. *Pathophysiology*.

[14] Yusipovich A. I., Braze N. A., Luneva O. G. (2013). Changes in the state of hemoglobin in patients with coronary heart disease and patients with circulatory failure. *Bulletin of Experimental Biology and Medicine*.

[15] Revin V. V., Gromova N. V., Revina E. S. (2015). Study of the structure, oxygen-transporting functions, and ionic composition of erythrocytes at vascular diseases. *BioMed Research International*.

[16] Nikinmaa M. (1997). Oxygen and carbondioxide transport in vertebrate erythrocytes: an evolutionary change in the role of membrane transport. *Journal of Experimental Biology*.

[17] Jewell S. A., Petrov P. G., Winlove C. P. (2013). The effect of oxidative stress on the membrane dipole potential of human red blood cells. *Biochimica et Biophysica Acta—Biomembranes*.

[18] McPherson R. A., Pincus M. R. (2011). *Clinical Diagnosis and Management by Laboratory Methods*.

[19] Brazhe N. A., Abdali S., Brazhe A. R. (2009). New insight into erythrocyte through in vivo surface-enhanced Raman spectroscopy. *Biophysical Journal*.

[20] Mityanina V. A., Parshina E. Y., Yusipovich A. I., Maksimov G. V., Selischeva A. A. (2012). Oxygen-binding characteristics of erythrocyte in children with type I diabetes mellitus of different duration. *Bulletin of Experimental Biology and Medicine*.

[21] Vlasov A. P., Trofimov V. A., Tarasova T. V. (2012). Structural-functional state of hemoglobin in gestosis. *Modern Problems of Science and Education*.

[22] Brazhe A. R., Brazhe N. A., Sosnovtseva O. V., Pavlov A. N., Mosekilde E., Maksimov G. V. (2009). Wavelet-based analysis of cell dynamics measured by interference microscopy. *Computer Research and Modeling*.

[23] Minaev V. L., Yusipovich A. I. (2012). Medical and biological measurements: use of an automated interference microscope in biological research. *Measurement Techniques*.

[24] Revin V. V., Filatova S. M., Syusin I. V. (2015). Study of correlation between state and composition of lipid phase and change in erythrocytes structure under induction of oxidative processes. *International Journal of Hematology*.

[25] Yusipovich A. I., Parshina E. Y., Brysgalova N. Y. (2009). Laser interference microscopy in erythrocyte study. *Journal of Applied Physics*.

[26] Tychinskiǐ V. P., Kufal' G. É., Vyshenskaya T. V., Perevedentseva E. V., Nikandrov S. L. (1997). Measurements of submicron structures with the Airyscan laser phase microscope. *Quantum Electronics*.

[27] Tychinskiï V. P. (2001). Coherent phase microscopy of intracellular processes. *Physics-Uspekhi*.

[28] Brazhe N. A., Brazhe A. R., Pavlov A. N. (2006). Unraveling cell processes: interference imaging interwoven with data analysis. *Journal of Biological Physics*.

[29] Yusipovich A. I., Zagubizhenko M. V., Levin G. G. (2011). Laser interference microscopy of amphibian erythrocytes: impact of cell volume and refractive index. *Journal of Microscopy*.

[30] Schindelin J., Arganda-Carreras I., Frise E. (2012). Fiji: an open-source platform for biological-image analysis. *Nature Methods*.

[31] Levin G. G., Bulygin Th. V., Vishnyakov G. N. (2005). Coherent oscillations of the molecular state of protein in live cells. *Tsitologiya*.

[32] Rappaz B., Barbul A., Charrière F. Erythrocytes volume and refractive index measurement with a digital holographic microscope.

[33] Mazeron P., Didelon J., Muller S., Stoltz J.-F. (2000). A theoretical approach of the measurement of osmotic fragility of erythrocytes by optical transmission. *Photochemistry and Photobiology*.

[34] Geary R. C. (1947). Testing for normality. *Biometrika*.

[35] Tukey J. W. (1949). Comparing individual means in the analysis of variance. *Biometrics. Journal of the Biometric Society*.

[36] Walker H. K., Hall W. D., Hurst J. W. (1990). *Clinical Methods: The History, Physical, and Laboratory Examinations*.

[37] Mohandas N., Clark M. R., Jacobs M. S., Shohet S. B. (1980). Analysis of factors regulating erythrocyte deformability. *Journal of Clinical Investigation*.

[38] Revin V. V., Gromova N. V., Revina E. S. (2016). Role of membrane lipids in the regulation of erythrocytic oxygen-transport function in cardiovascular diseases. *BioMed Research International*.

[39] Boveris A., Chance B. (1973). The mitochondrial generation of hydrogen peroxide: general properties and effect of hyperbaric oxygen. *Biochemical Journal*.

[40] Toschi V., Gallo R., Lettino M. (1997). Tissue factor modulates the thrombogenicity of human atherosclerotic plaques. *Circulation*.

[41] Perticone F., Ceravolo R., Pujia A. (2001). Prognostic significance of endothelial dysfunction in hypertensive patients. *Circulation*.

[42] Fornal M., Korbut R. A., Lekka M. (2008). Rheological properties of erythrocytes in patients with high risk of cardiovascular disease. *Clinical Hemorheology and Microcirculation*.

[43] Jiménez J. A., Loango N., Giraldo A. M., Landázuri P., Castaño H. (2012). Sphingomyelin of erythrocytes membranes is related to total cholesterol and LDL-cholesterol in patients with significant coronary arterial disease. *The Open Clinical Chemistry Journal*.

[44] Saldanha C., Sargenter L., Monteiro J., Perdigão C., Ribeiro C., Martins-Silva J. (1999). Impairment of the erythrocyte membrane fluidity in survivors of acute myocardial infarction. A prospective study. *Clinical Hemorheology and Microcirculation*.

[45] Levine A. B., Punihaole D., Levine T. B. (2012). Characterization of the role of nitric oxide and its clinical applications. *Cardiology*.

[46] Hille R., Olson J. S., Palmer G. (1979). Spectral transitions of nitrosylhemes during ligand binding to hemoglobin. *The Journal of Biological Chemistry*.

